# ipd: an R package for conducting inference on predicted data

**DOI:** 10.1093/bioinformatics/btaf055

**Published:** 2025-02-03

**Authors:** Stephen Salerno, Jiacheng Miao, Awan Afiaz, Kentaro Hoffman, Anna Neufeld, Qiongshi Lu, Tyler H McCormick, Jeffrey T Leek

**Affiliations:** Public Health Sciences, Biostatistics, Fred Hutchinson Cancer Center, Seattle, WA 98109, United States; Biostatistics and Medical Informatics, University of Wisconsin-Madison, Madison, WI 53726, United States; Public Health Sciences, Biostatistics, Fred Hutchinson Cancer Center, Seattle, WA 98109, United States; Biostatistics, University of Washington, Seattle, WA 98195, United States; Statistics, University of Washington, Seattle, WA 98195, United States; Mathematics and Statistics, Williams College, Williamstown, MA 01267, United States; Biostatistics and Medical Informatics, University of Wisconsin-Madison, Madison, WI 53726, United States; Statistics, University of Washington, Seattle, WA 98195, United States; Sociology, University of Washington, Seattle, WA 98195, United States; Public Health Sciences, Biostatistics, Fred Hutchinson Cancer Center, Seattle, WA 98109, United States; Biostatistics, University of Washington, Seattle, WA 98195, United States

## Abstract

**Summary:**

ipd is an open-source R software package for the downstream modeling of an outcome and its associated features where a potentially sizable portion of the outcome data has been imputed by an artificial intelligence or machine learning prediction algorithm. The package implements several recent proposed methods for inference on predicted data with a single, user-friendly wrapper function, ipd. The package also provides custom print, summary, tidy, glance, and augment methods to facilitate easy model inspection. This document introduces the ipd software package and provides a demonstration of its basic usage.

**Availability:**

ipd is freely available on CRAN or as a developer version at our GitHub page: github.com/ipd-tools/ipd. Full documentation, including detailed instructions and a usage ‘vignette’ are available at github.com/ipd-tools/ipd.

## 1 Introduction

With the rapid advancement of artificial intelligence and machine learning (AI/ML) algorithms, and owing to financial and domain-specific constraints, researchers from a wide range of disciplines increasingly use predictions from pre-trained algorithms as outcome variables in statistical analyses (Hoffman *et al.* 2024). However, reifying algorithmically derived values as measured outcomes may lead to potentially biased estimates and anti-conservative inference (e.g. see, Wang *et al.* 2020). In particular, the statistical challenges encountered when drawing *inference on predicted data* (IPD) include: (i) understanding the relationship between the predicted outcomes and their true, unobserved counterparts, (ii) quantifying the robustness of the AI/ML models to resampling or uncertainty about the data they were trained on, and (iii) appropriately propagating both bias and uncertainty from upstream predictive model into downstream inferential procedures. We refer to methods developed to address these challenges as methods for conducting IPD.

Several recent methods have been proposed for conducting IPD. These include *post-prediction inference (PostPI)* by Wang *et al.* (2020), *prediction-powered inference (PPI)*, and *PPI++* by Angelopoulos *et al.* (2023a,b), and *post-prediction adaptive inference (PSPA)*, as well as PSPA’s extensions, *POP-TOOLS* and *post-prediction sumstats-based inference (PSPS)* by Miao *et al.* (2023, 2024) and Miao and Lu (2024), respectively, *prediction-powered bootstrap (PPBoot)* by Zrnic (2024), and semi-supervised methods, *cross-prediction-powered inference (Cross-PPI)* by Zrnic and Candès (2024) and *design-based supervised learning (DSL)* by Egami *et al.* (2023). In general, these methods employ one of two strategies for IPD correction. They either (i) construct pseudo-outcomes designed to resemble the true, unobserved outcome and correct inference on the outcome space, or (ii) calibrate the parameter estimates and standard errors directly.

These methods have been developed in quick succession in response to the ever-growing practice of using predicted data directly to conduct statistical inference. To enable researchers and practitioners interested in fully using these state-of-the-art methods, we have developed ipd, a comprehensive open source software package that implements these existing methods under the umbrella of IPD. Moreover, we provide a convenient wrapper and helper functions, so these methods can be compared in numerous inferential settings. This note provides an overview of the package, including installation instructions, basic usage examples, and additional documentation. The examples presented here show how to generate data, fit models, and use custom methods provided by the package.


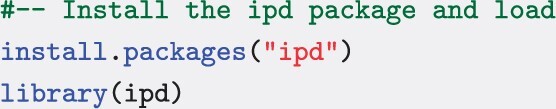


## 2 Installation and usage

ipd is implemented in the R statistical computing language (R Core Team 2024). Full documentation, including detailed downloading and installation instructions and usage ‘vignettes’ are available on the package website: github.com/ipd-tools/ipd. The ipd package has been successfully tested and runs on all the latest versions of Windows, Mac OS X, Ubuntu (Linux) operating systems. To install the development version of ipd from GitHub, one can use the devtools package.

We provide a simple example to demonstrate the basic use of this function. Following the notation of Miao *et al.* (2023), we assume the user has a dataset, D=L ∪ U, consisting of $L=\{(Y_{i},f_{i},X_{i},Set_{i});\;i=1,\ldots ,n\}$  *labeled* samples of the observed outcome, Y, predicted outcome, f, and features of interest, X, and $U=\{(f_{i},X_{i},Set_{i});\;i=n+1,\ldots ,n+N\}$  *unlabeled* samples, where the outcome is not observed. Set is a variable indicating whether the ith observation is *labeled*, where $Set_{i}=1$ if the observation is *labeled* and $Set_{i}=0$ otherwise.

The main function, ipd, gives access to the various methods for conducting IPD with different potential estimands. The user supplies a formula of the form Y−f = X1 + X2 + …, where Y is replaced by the name of the observed outcome variable in the data, f is replaced by the name of the predicted outcome, and X1, X2, …, are the names of the independent variables (features) of interest. The user has the option to supply either a stacked dataset and the name of the column corresponding to the Set label indicator, via the data and label arguments, respectively, or to supply labeled and unlabeled data sets separately, via the data and unlabeled_data arguments, respectively. Not all columns in data or unlabeled_data may be used unless explicitly referenced in the formula argument or in the label argument. Options for the available methods, specified via the method argument, include “postpi_analytic” or “postpi_boot” (Wang *et al.* 2020), “ppi” (Angelopoulos *et al.* 2023a), “ppi_plusplus” (Angelopoulos *et al.* 2023b), and “pspa” (Miao *et al.* 2023). Current estimands, specified via the model argument, include the population mean (“mean”) or qth quantile (“quantile”, with additional argument q = q) of the outcome, linear (“ols”), and logistic (“logistic”) regression. Future development will include a broader class of exponential family model parameters including multiclass logistic regression, time-to-event models, and causal targets such as average treatment effects (ATE). Additional (optional) arguments include the significance level, alpha, for the 100(1−α)% confidence intervals, and other method-specific arguments.

The function outputs a “glm”-style list with the parameter estimates, standard errors, and confidence limits, as well as additional information about the function call and intermediate estimated quantities [e.g. the estimated relationship model of Wang *et al.* (2020) or the estimated tuning parameters of Angelopoulos *et al.* (2023a,b) and Miao *et al.* (2023)].

All method-specific and helper functions are documented and exported by the ipd package for additional user flexibility. These include a function to generate simulated data, simdat, to facilitate exploration of the methods in the absence of real data, as well as print, summary, tidy, glance, and augment methods to facilitate easy model inspection (Wickham *et al.* 2023). Documentation for the package and these functions can be accessed by running ?ipd-package, ?<function> (e.g., ?simdat) or help(“simdat”) in the R console. In the next section, we provide an illustrative example using simulated data generated by simdat for linear regression.

## 3 An example analysis

We simulate a continuous outcome for linear regression, with $Y=\beta_{1}X_{1}+\frac{1}{2}X_{2}^{2}+\frac{1}{3}X_{3}^{3}+\frac{1}{4}X_{4}^{2}+\varepsilon ,$ where $X_{1}$, $X_{2}$, $X_{3}$, and $X_{4}∼N(0,1)$, $\beta_{1}=1$, corresponding to the true value of the linear regression coefficient for $X_{1}$ (our target of inference), and $\varepsilon ∼N(0,\sigma_{Y}^{2});\sigma_{Y}=4$ using the simdat function. We generate a stacked dataset of 100 training, 100 labeled, and 1000 unlabeled observations, where the corresponding set is denoted by a column called “set_label.” We generate predicted outcomes by training a generalized additive model on the training set and making predictions for the labeled and unlabeled sets.


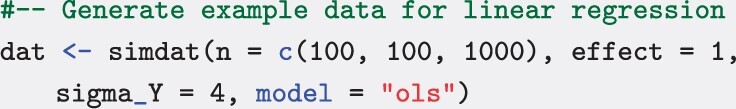


For each method, we calculate the point estimate and corresponding 100(1−α)% confidence interval for $\beta_{1}$, where α=0.05 and we have a two-sided hypothesis test. We compare the methods in the package to three additional models, which serve as performance benchmarks: the “oracle” regression, which fits the ideal model on the true, unknown outcome for the unlabeled observations (possible on simulated data), the “naive” regression, which treats the predicted outcomes as if they were the true, unobserved outcomes, and the “classic” regression, which utilizes only the labeled subset of the data:


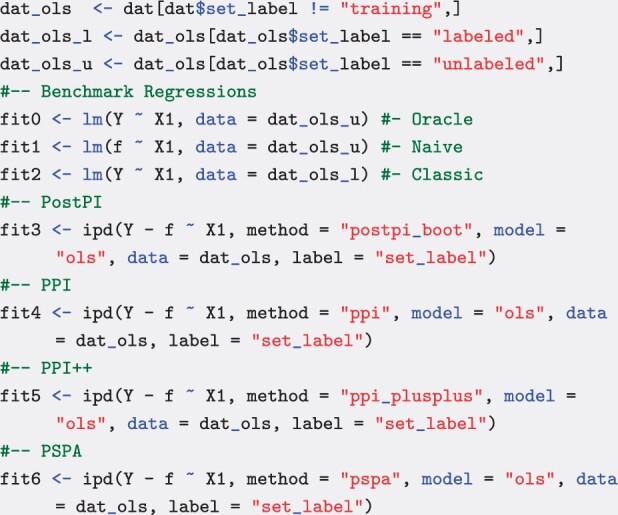


**Figure 1.**
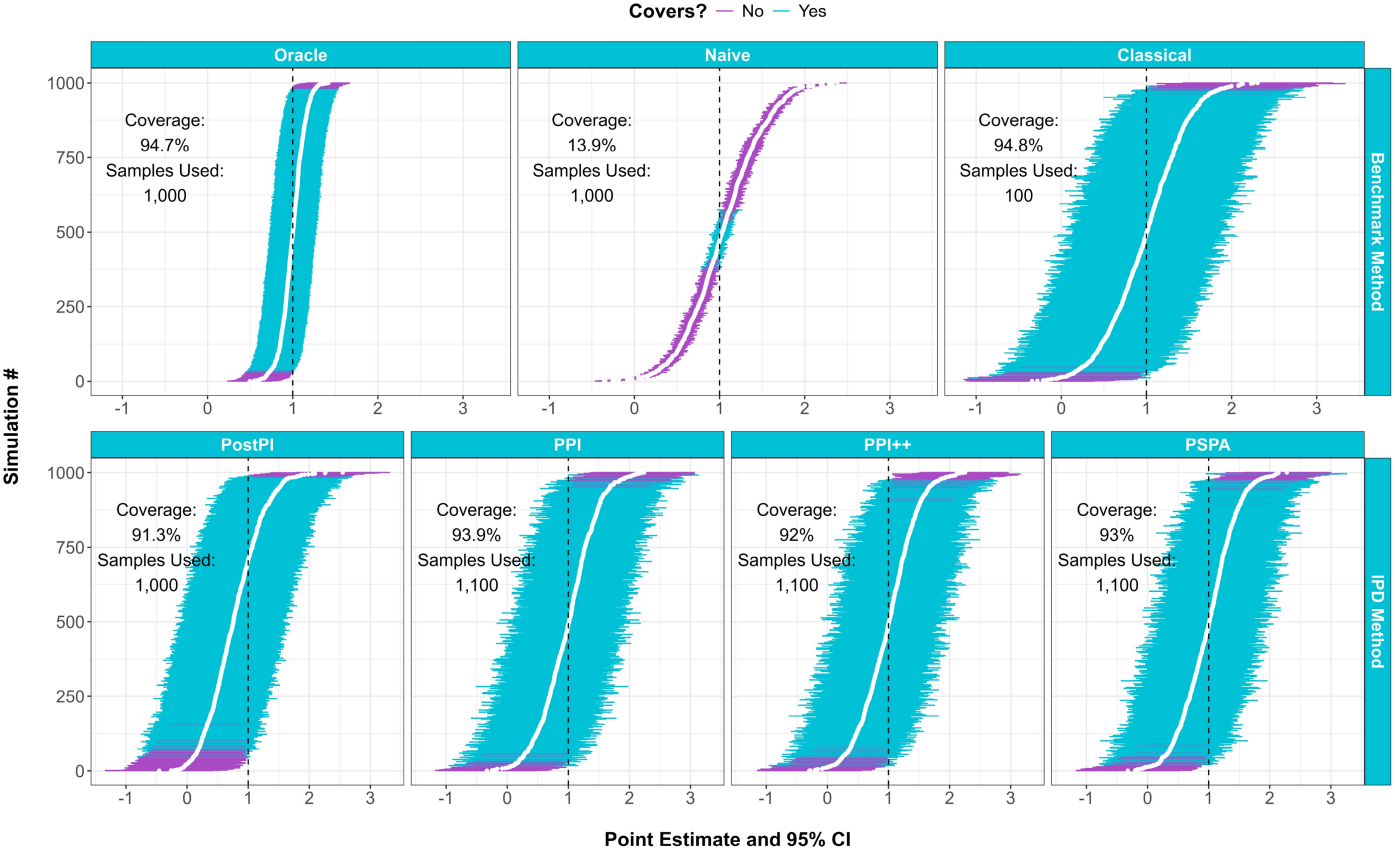
Point estimate and corresponding 95% confidence intervals for four available IPD methods (postpi, ppi, ppi_plusplus, and pspa; second row), as compared to three benchmark regressions (oracle, naive, and classical; first row) on 1000 simulated linear regression datasets.

As a benchmark, the hypothetical ‘oracle’ regression would have the correct estimate and correctly-sized confidence interval if the outcome was measured for each observation, while in practice, the “naive” regression may have a biased point estimate and a confidence interval that is too narrow. The “classical” regression will have the correct estimate but wider confidence interval, as it only uses the labeled subset of the data. The implemented IPD methods all correctly estimate the coefficient and have confidence intervals that are wider than the “oracle” but narrower than the “classic” regression (Fig. 1).

## 4 Printing, summarizing, and tidying

The package also provides custom print, summary, tidy, glance, and augment methods to facilitate easy model inspection. Namely, the print method gives an abbreviated summary of the output from the ipd function, the summary method gives more detailed information about the estimated coefficients, standard errors, and confidence limits, The tidy method organizes the model coefficients into a “tidy” format (Wickham *et al.* 2023), the glance method returns a one-row summary of the model fit, and the augment method adds model predictions and residuals to the original dataset. For a more detailed look into using the ipd wrapper function and the method-specific individual functions, please refer to the vignettes provided with the package.


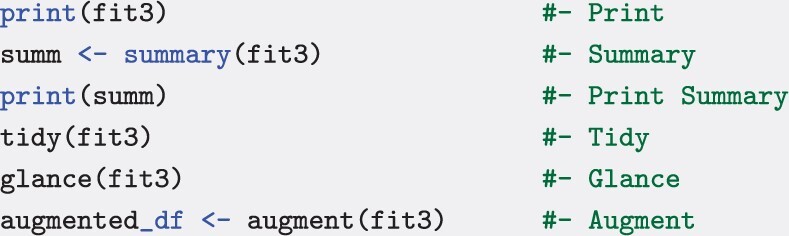


## 5 Conclusion

In this note, we present ipd, a comprehensive R package which implements various recent methods for conducting inference on predicted data. We highlight the usability of this software for practitioners to draw valid inference on algorithmically derived outcomes, as well as the ability to facilitate comparisons for those wishing to further develop methods in the space of IPD. It is our hope that we, and others members of the research community, will maintain and grow this package as the field of IPD continues to mature in the current AI/ML era.

Conflict of interest: J.T.L. reports teaching Coursera courses which generate revenue for both Johns Hopkins University and the Fred Hutchinson Cancer Center. J.T.L. reports co-founding and serving on the board of Synthesize Bio.

## Data Availability

No new data were generated or analysed in support of this research.
